# Assessment of the impact of management commitment and supply chain integration on SMEs’ innovation performance: Moderation role of government support

**DOI:** 10.1016/j.heliyon.2023.e15914

**Published:** 2023-04-29

**Authors:** Hao Chen, Timothy Amoako, Collins Ewudzie Quansah, Stephen Abiam Danso, Dawud Jidda Jidda

**Affiliations:** a Jiangsu University, China; b Kwame Nkrumah University of Science and Technology, China

**Keywords:** Management commitment, Supply chain integration, Government support, SMEs' innovation performance

## Abstract

This study aims to investigate the effect of management commitment (MC), supply chain integration (SCI), and government support (PGS) on small and medium enterprises (SMEs) innovation performance. The study was quantitative, and the cross-sectional method was used to gather 685 valid data through a structured questionnaire. Confirmatory factor analysis in Analysis of Moment Structures version 26 software was engaged in checking the constructs' validity. Hierarchical regression analysis was employed to examine the hypothesized relationships using Statistical Package for Social Sciences version 26 software. The regression analysis findings revealed that management commitment influenced the three dimensions of SCI (internal, customer, and supplier integration) and SMEs' innovation performance. The results from the mediation analysis indicated a partial mediation effect of internal, customer, and supplier integration in the relationship between management commitment and SMEs' innovation performance. Also, PGS significantly moderated the relationship between SCI and SMEs' innovation performance. The present study is critical as it explains the medium through which MC, SCI, PGS, and SMEs’ innovation performance relate in one conceptual model.

## 1 Introduction

Innovation performance is the application of creativity or innovative ideas to enhance products, processes, and procedures to raise the importance, value, and performance of a product or service [1]. This requires converting innovation resources and competencies into outputs that achieve innovative market success [2]. The primary reason for promoting innovation is to boost a firms' internal growth and competitiveness [3]. Innovation lays the foundation for firms' improved performance, economic development, and countries' wealth [4]. Thus, high performance within small and medium enterprises (SMEs) depends on the firm's innovative capabilities, leading to higher innovation performance [5,6]. However, even though SMEs are often regarded as the backbone of most economies and account for more than 92% of all businesses in most nations [7], few have well-honed innovation processes embedded in their firms [8]. Hence, stagnate SMEs' operations and eventually loses their competitive edge [9]. Accordingly [10], emphasized that SMEs' must integrate and sustain innovation by creating a sense of purpose among teams, activating creativity, and identifying unusual opportunities. Therefore, in achieving sustained innovation, the current study assesses how management commitment through the mediating role of supply chain integration may contribute to high SMEs' innovation performance. The study also estimates how government support can moderate the impact of supply chain integration on SMEs' innovation performance.

Available literature acknowledges the vital role MC plays in developing the inner drive of a firm's workforce, enhancing SMEs' innovation performance [11,12]. Studies have reported a positive influence of management commitment on firm performance [[13], [14], [15]]. These studies' findings suggest that the positive relationship arises from instances where other mechanisms may contribute to MC's influence on performance. For example [[14], [15]], substantiated this mechanism by assessing customer relationship management as a mediating variable between management commitment and business performance. Also [[13], [14]], established how just-in-time partially mediated the relationship between management commitment and operational performance.

Furthermore, studies have recognized commitment as a prerequisite for attaining full supply chain integration (SCI) in firms [[15], [16], [17]]. [18,19] indicated that commitment is vital for successfully implementing SCI. Again [20], emphasizes that when commitment is motivated in a particular firm, it leads that firm to work cooperatively with other firms to implement SCI. Studies such as [[21], [22], [23]] have indicated the positive influence of SCI on firm performance. The studies’ results emphasize that effective SCI produces higher performance. In other words, firms could gain a competitive advantage by effectively integrating with supply chain partners to reduce production costs, improve product quality, shorten cycle time, increase response rates, and improve customer satisfaction [24,25]. Fundamentally, the key to SCI is establishing constant connections with downstream customers and upstream suppliers and a complete internal functional synergy [24]. Accordingly [[24], [25]], argues that despite the growing academic interest in SCI, there is limited understanding of what influences MC leading to higher performance. In this regard, we include SCI as the mediating variable and provide empirical evidence for the connection.

The study also assessed how the mediating mechanism (SCI) might change as a function of other factors like government support (PGS). PGS refers to how central or local government assists firms, such as implementing programs or policies in a particular country or region [[26], [27], [28], [29]]. Studies have indicated that government support to firms improves innovation in firms leading to higher performance [[30], [31]]. In other words, PGS offers financial or technical support and reshapes management views on innovation [29]. By this, PGS, as a resource to an internally integrated firm, will aid in realizing effective and efficient external integration [[32], [33]]. PGS, such as tax credits, loans, policies, subsidies, research, and development, could improve a firm's internal structures and networked external partners [34]. Equally, SMEs engaged in external integration can leverage on PGS to enhance their business activities and achieve higher innovation performance. Studies such as [29,32] have extensively researched PGS and emphasized that government support benefits firms and boosts employees' confidence. Despite PGS's importance, its moderation effect in the relationship between SCI and SMEs' innovation performance is not established in the literature. Hence, we propose that PGS could further strengthen SCI's effects on innovation performance.

To address the lacunae in the literature, we relied on the dynamic capability view (DCV) in developing our hypothesized relationships. [[35], [36]] indicated that firms that possess solid dynamic capabilities assist managers in learning from inside and outside the firm, integrating acquired resources, and adapting appropriately to external developments. This results in value creation and innovation in firms leading to higher performance. Considering this, the current study's theoretical and practical contributions fulfill three objectives: (i) to identify whether MC and SMEs' innovation performance that has been generally studied in Asian and Western large firms [17,25,37] is also applicable in the framework of small and medium enterprises in a developing economy such as Ghana; (ii) to ascertain a clear understanding of how MC influences SMEs' innovation performance via a mediating mechanism of SCI; and (iii) to encourage SMEs' to integrate their internal structures and effectively link up with external partners to attract external resources such as PGS and improve SMEs' innovation performance.

The remainder of the study is organized as follows: Section 2 deals with the theoretical foundation and literature review. Further, the study's conceptual framework is developed based on the literature reviewed. Then, sections 3, 4, 5 describe the methodology, results, and discussion, respectively, while section 6 discusses theoretical and managerial contributions. Finally, section 7 presents the limitations of the study.

## 2 Theoretical foundation and literature review

### 2.1 Dynamic capability view

Dynamic capability view (DCV), which emerged from the resource base view (RBV), is the ability of firms to establish, integrate, and reconfigure internal and external resources to attain sustained competitive advantages [[38], [39], [40]]. The DCV espouses how intangible assets, capabilities, and internal and external resources have become the most valuable assets for firms to achieve competitive advantage [36]. Thus, firms can rely on DCV to create unique intangible assets such as management commitment [41]. Studies have indicated that MC influences performance [14,42]. In effect, DCV will aid committed managers to develop effective capabilities to efficiently coordinate teams, establish a peculiar culture that encourages and support innovation and adapt to changes [43,44]. Based on the proposition of DCV, committed managers will be able to sense, seize and reconfigure resources [36] by integrating a firm's internal aspect into a synchronized framework and further link with external partners such as customers and suppliers [33]. This causes MC to have the potency to influence SCI [24] and, consequently, improve innovation performance among SMEs [16,17].

Additionally, for SMEs' to reconfigure their resources and achieve a sustainable competitive advantage, firms must complement SCI with external resources such as government support (PGS) [45]. As argued by Refs. [[28], [29], [46]], PGS improves innovation in firms. Therefore, firms leveraging PGS can influence their SCI to maximize SMEs’ innovation performance.

### 2.2 Management commitment and innovation performance

Innovation performance in this study is based on SMEs’ ability to advance new products or services quickly, introduce new products or services on time, develop changes in business processes, and swiftly respond to new strategies presented by competitors in the industry [[46], [47]].

Regardless of the firm's size, innovation mostly begins with innovative ideas from managers [[40], [47],^48^]. Manager-driven innovation is the creation and execution of new ideas, products, or processes that stem from a single manager's efforts or the entire management team [5]. Consequently, a manager's highly sophisticated and tacit knowledge is vital in gaining a competitive advantage [38].

According to Ref. [49], management refers to a person or group with the power to direct and oversee management, establish vision and goals, formulate rules and policies, allot resources, and carry out projects. Therefore, management commitment (MC) is an emotional attachment and loyalty demonstrated by managers who dedicate their ideas, attention, and responsibilities to achieving the firm's missions, values, and goals [50]. In this vein, committed managers possessing dynamic capabilities are seen to allot resources and time to the firm's management, empower, support employees, and supervise operations to achieve firm objectives [13,14,37]. For instance Ref. [51], postulated that higher management commitment serves as a governing mechanism in the organization and allows for the development of firm capacity by enhancing employees' capabilities. The researchers further indicated that management commitment is essential for achieving long-term sustainable performance in the firm. From the standpoint of DCV, it could be argued that MC is critical in reconfiguring firm resources, developing capabilities, and assisting in gaining a competitive advantage [52], leading to higher innovation performance. Studies such as [[12], [13], [14], [16]] have found a positive influence of management commitment on firm performance. For instance Ref. [[16], [17]], indicated a positive effect of employee commitment on operational performance. The researchers posit that a firm having committed employees could be the foundation for developing firm capabilities, which can generate a competitive advantage. Last [[12], [13]], examined the effects of organizational commitment on innovation performance from employees' perspectives. The study findings revealed that emotional attachment and belongingness improve employees' commitment to their firm. The results verified MC's influence on innovation performance. From the arguments enumerated, it is evident that MC significantly influences SMEs' innovation performance. However, the literature that empirically proposes how MC affects SMEs' innovation performance is insufficient. Therefore, we propose that:

H1 Management commitment positively influences SMEs' innovation performance.

### 2.3 Effects of management commitment on supply chain integration

Management commitment (MC) refers to how management supports and participates in the firm's strategy and operations [37]. In this sense, management establishes and communicates the firm's vision and goals, involves and participates in the firm's management, allots resources and time, and motivates and empowers employees to internalize firm values and monitor operations [50,53] to achieve effective supply chain integration (SCI). For instance Ref. [54], argued that firms with total commitment, values, and norms are well informed by the staff and are inherently tight to established goals, hence becoming aware of the importance of SCI strategies. Primarily, committed managers with dynamic capabilities will likely exhibit the utmost desire to engage in the firm's activities, including SCI practices.

According to Ref. [25], the influence of management commitment on SCI can be expounded on three main principles: communication, strategic partnership, and working together. Researchers emphasized that committed manager recognizes the firm's partnership strategy as their priority. Again, committed managers are prepared to share acquired knowledge, resources, support, and enhance communication among employees in different departments to benefit external partners [55]. [56] also argued that committed managers shred away individual attitudes and work collectively to improve firm performance.

Conferring from the dynamic capability view (DCV), internal integration (INT) could be seen as the internal capability of the firm [36]. Thus, committed managers with dynamic capabilities engage in cross-functional partnerships that help formulate functional strategies and practices into synchronized processes [57]. These processes could lead to the build-up of the firm's unique capabilities. The acquired unique capabilities could aid a firm to compete by using the internal capabilities to access external opportunities, identify threats and address internal weaknesses [58]. A study by Ref. [25] emphasized that committed managers with strong motivation for the firm goals and an understanding of SCI strategies will improve communication and partnerships with internal functions.

External integration, which is made up of customer (CIN) and supplier integration (SI) could be argued as an external capability of a firm [59,60]. Thus, Committed managers who initiate customer integration (CIN) will promptly communicate firm strategies and operational activities to customers and deal with customer requirements [57]. This will help improve the performance within the supply chain. The study of [37] found that management commitment positively influences customer integration. The findings demonstrate that management commitment is critical in directing the firm to care for its customers. Again, a study conducted in the Chinese automobile industry indicated that management commitment is essential in maintaining positive supplier relationships [61]. Hence, committed managers’ efforts to share financial, operational, and strategic knowledge and data with suppliers reduce inventory and supplier lead time [62].

Consequently, this will lead to a strategic alliance between the firm and its suppliers that will benefit both parties [63]. Furthermore, studies such as [61,64] found a positive influence of management commitment on SI. Therefore, we propose that:

H2a Management commitment positively influences INT.

H2b Management commitment positively influences CI.

H2c Management commitment positively influences SI.

### 2.4 Effects of SCI on innovation performance

From the dynamic capability view, a firm may be able to modify its supply chain management capabilities to align better with supply chain objectives [24,65]. This includes having a capable supply chain integration that can significantly stand the test of time and contribute to innovative performance. Thus, firms could acquire dynamic capabilities through SCI [65]. The acquired capabilities allow firms to attain and absorb external knowledge and develop strategic routines for supply chain partners in the diverse competitive business environment [66]. Again, DCV elucidates how internal and external integration strategic alliance is critical in improving a firm's dynamic capabilities [55].

Internal integration (INT) is the ability to structure a firm's functional practices, strategies, and processes into synchronized and collaborative processes [55]. INT highlights the importance of different functional departments of a firm operating as an integrated process rather than isolated silos, which is made possible with enterprise resource planning [67]. When functional barriers are curtailed, effectiveness and efficiency are achieved in the firm's internal operations leading to higher innovation performance. Collaboration among functional units helps break silos connected with traditional departmentalization and accelerate firm conflict resolution and response [67,68]. The coordinated functional units focus on attaining collective goals and enhancing the efficient usage of firm resources [69], positively affecting a firm's innovation performance. Studies such as [67,70] have verified the positive predictive effect of INT on firm performance. Both studies argued that INT is a precondition for external integration. Therefore, we propose that:

H3a Internal integration has a positive predictive effect on innovation performance.

Customer integration (CIN) refers to a firm's ability to arrange its inter-firm strategies, procedures, practices, and behavior into synchronized and well-established processes to achieve consumer needs [71]. It assists customers in the supply chain to establish strategic partnerships that improve their competitiveness at a low transactional cost [57], leading to more significant market opportunities that could improve a firm's innovation performance. Close coordination with customers via collaborative planning, real-time data sharing, and jointly managed inventories aid firms in responding quickly to market requirements in an unstable business environment [72]. CIN denotes the mutual collaboration of customers with the focal firm in strategically sharing information and experience about demands and performance, such as revenue, sales volume, and customer satisfaction [[25], [26]]. Studies such as [67,73] have established a positive predictive effect of CIN on firm performance. Both studies postulated that their results support that customers are the bases of information for the demand market. Hence, poor connection with key customers would slow access to market information. Therefore, we propose that:

H3b Customer integration has a positive predictive effect on innovation performance.

Supplier integration (SI) is the degree to which practices among firms and their strategic suppliers enable the efficient transfer of resources and expertise, which is critical for generating mutual gains [74]. It requires closer alliance and coordination with major suppliers to ascertain shared benefits such as supplier lead time and reduction in inventory [75]. SI is the most common kind of SCI [76] which improves innovation performance. Thus, it is argued that establishing long-term and close relationships with significant suppliers reduces opportunistic behaviors and irregularities in transactions, improves product quality, and decreases expenditure on monitoring, delivery, and performance [77]. Suppliers have also assumed the role of strategic collaborators in the integration process, allowing the focal firm to access their technological and operational resources [[16], [17]], which helps in product design and development.

Similarly, the focal firm must communicate with key suppliers and regularly update data gathered in the integration process to inform partners of changes in the business environment. Studies conducted by Refs. [67,78] established a positive predictive effect of SI on firm performance. Both studies stressed the need to develop effective SI via timely data exchange between the focal firm and suppliers. Therefore, we propose that:

H3c Supplier integration has a positive predictive effect on innovation performance.

### 2.5 Mediation role of supply chain integration

Management commitment (MC) offers a linchpin for executing SCI [25]. This indicates that MC promotes the firm's internal structure to advance strategic partnerships, communication, support, and working together [79,80]. Consequently, committed managers of a firm may recognize the firm's strategy of entering into a partnership as their priority and are likely to share their expertise and knowledge [81]. Again, committed managers will shred away their interests, allot resources, cooperate and motivate employees, and work effectively in the greatest interest of the firm. For internal integration (INT), committed managers would thus engage across diverse functions, tactically collaborate, and work together in the integrated process to maximize performance [75].

From the dynamic capability viewpoint (DCV), committed managers with a firm could relay the firm's strategies and operational requirements to customers and suppliers, engage suppliers in product designs, and reactively deal with customers' desires [69]. Additionally, committed managers will aspire to advance a long-term relationship with the attached firm suppliers, and customers through sharing resources. Again, an established close and long-term relationship between the focal firm, suppliers, and customers tends to reduce opportunistic behaviors, monitor costs, and surge product quality, delivery, and improve innovation performance [77]. Studies such as [14,82] have achieved a significant positive relationship between management commitment and firm performance. They suggest that commitment is a prerequisite for attaining integrated supply chain.

Supply chain integration (SCI) can be considered a strategic resource that leads to competitive advantages, according to the resource base view (RBV) [83]. In the extant literature, SCI (internal, customer, supplier) has been verified to influence firm performance [24,67,78]. Many studies have suggested that INT increases a firm's performance by allowing for better intra-firm coordination and collaboration among various departments [[40], [83]]. Sharing integrational data and cross-departmental collaboration could make this possible [75]. Furthermore [60], highlighted that firms must strive to integrate, adapt and reconfigure internal and external integrative capabilities to meet the demands of a turbulent business environment. In this vein, achieving high innovation performance is possible if focal firms build a solid relationship with external partners and then penetrate deep into the external firms to gain access to information and resources valuable to the focal firm. Such actions will allow the focal firm to effectively and efficiently respond to external partners' demands. Thus, when this collaborative framework is well developed, it could result in advantages, such as reduced inventory or decreased supplier lead time [[83], [84]]. Customer integration (CIN) helps appreciate significant customer demands and considerations in the firm's processes [[83], [84]], positively impacting performance. Per available literature, it could be argued that MC forms the foundation for effective SCI; SCI will also, in turn, influence SMEs' innovation performance. The effects of MC on SMEs' innovation performance could be actualized via effective SCI. Therefore, from the standpoint of DCV, the current study examines the mediation role of SCI in the relationship between MC and SMEs' innovation performance. Hence, we propose that:

H4a INTI mediates the relationship between MC and innovation performance

H4b CI mediates the relationship between MC and innovation performance

H4c SI mediates the relationship between MC and innovation performance.

### 2.6 Moderation role of government support

From a broader perspective, the dynamic capability view (DCV) postulates that capabilities that create a firm's competitive advantage are partly achieved through integrating internal and external resources [36,85]. The developed capabilities become challenging to imitate and duplicate. Therefore, firms must effectively leverage their external resources, such as government support (PGS) [29], to pursue core capabilities and sustain their competitive advantage.

Government supports SMEs' innovation through local, regional, and national programs [30]. Accordingly, support services the government provides to improve SME innovation performance include: cutting administrative costs and burdens, building networks across sectors and borders, technical and managerial training programs, legal framework reinforcement, and provisions for financial assistance [30]. Arguably, the rationale behind government support (PGS) for SMEs' includes, in the situation where there is market failure or disruption, bringing bias against SMEs' [30]. Additionally, Small size tends to create cost disadvantages and limit development capabilities. Such restraints raise SMEs' transactional costs and impede SMEs’ ability to take advantage of economic opportunities, granting the need for PGS.

Resource dependence theory (RDT) posits that inter-firm strategies are undertaken to mitigate the adverse effects of external pressures and improve a firm's strategies [[86], [87]]. Consistently, the literature suggests that strategic alliances (supply chain integration) and networks (with the government) to seek support are essential extra-firm strategies to secure resources and influence [27]. Again, PGS is acknowledged as a crucial driver for the firm's survival and progress, even though it does not considerably contribute to its profitability [[87], [88]]. Hence, the government's support could influence the firm's internal structures to build the firm's resources and capabilities. In addition, the resources and capabilities acquired through PGS could create experience and knowledge beyond the confines of a functional area or a single department to assist external integration, which will, in turn, facilitate goal alignment and boost performance [22].

Internal integration (INT) is a prerequisite for external integration that is supplier and customer [[25], [26]]. External integrational activities with support from the government could reduce long-term planning and increase demand forecasting. In effect, PGS (technical training, credit, loans, services, tax waivers, network building, research, and development) in suppliers' operations could enhance suppliers' understanding of the manufacturers’ requirements and assist the manufacturers in improving services for their customers [65]. This, in turn, maximizes innovation performance.

Customer integration (CIN) is an integral part of external integration, which is significant in appreciating the focal firm's clients' requirements [89]. Customers are primarily noted as the lifeline for most firms [[77], [90]]. CIN accomplishments with support from government aid in identifying, understanding, and exploiting clients' demands to manufacture customer-focused products to increase customer satisfaction [78]. Thus, PGS as a policy is perceived to influence SCI (internal, customer, and supplier) and yield higher SMEs' innovation performance.

Studies such as [[29], [30], [31]] have suggested that PGS could strengthen firms’ innovation or performance through tax waivers, credits, loans, and subsidies. More so, in the study of [29], PGS assumed a moderation role that further strengthened the positive impact of innovative climate on department innovations. Therefore, researchers propose that:

H5a PGS will significantly and positively moderate the relationship between INT and innovation performance.

H5b PGS will significantly and positively moderate the relationship between CIN and innovation performance.

H5c PGS will significantly and positively moderate the relationship between SI and innovation performance.

**Fig. 1:**
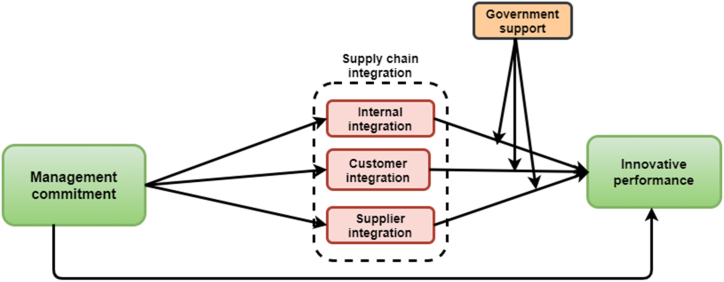
A proposed conceptual framework.

The study's conceptual framework is depicted in Fig. 1. The proposed framework details the connections between the factors examined.

## 3 Methodology

### 3.1 Participant, data collection, and response pattern

The research participants were managers of small and medium enterprises (SMEs) registered under the Ghana Enterprise Agency (GEA), [[90], [91]]. The service and manufacturing SMEs' are the two main most significant contributors to the economy of Ghana and employ over 85% of the working population [7]. Hence, worth studying. This research used the “employee number” criterion advocated by GEA in defining SMEs. The criterion stipulates that firms with 6–29 employees are classified as small, and 30–99 employees are classified as medium enterprises. After obtaining a list of registered SMEs from GEA, including their names and nature of businesses, year of registration, contact information, and location, we conducted a pilot study. About 75 general managers of SMEs’ in Accra, the capital city of Ghana, were used for the pilot testing.

The reliability of the pilot-tested items was good. Therefore, we proceeded to administer the questionnaire to a large sample. Before issuing the questionnaires to a large sample between the periods of (October 2021 and November 2021), we purposively sent emails to the list of firms we had obtained from the GEA, informing them about the objective of our study. And that we would require them to respond to some survey questions on SMEs. Those we could not hear from were contacted through telephone calls. We also visited the companies if no one responded to our telephone calls. We undertook these exercises to increase the number of participations in our survey. Unfortunately, we could not reach all the firms. Some firms we visited on more than three occasions were never opened. A possible reason could be that the Covid-19 pandemic, which disrupted the supply chain process, might have affected these small businesses, as the owners stayed home for a long time due to the Covid lockdowns [[91], [92]]. Their working capital might have been affected during the long period they stayed home as they had to depend on their available money for survival. Our inability to reach some SME owners and managers could have affected our study's sample size. However, we made several attempts to increase participation. We encouraged the ones we could reach to complete the questionnaires honestly. We indicated to the respondents that there was no right or wrong answer. Also, we promised them of utmost confidentiality and privacy.

Overall, 700 questionnaires were issued to managers, and 685, representing 97.86%, were returned to the authors as valid responses. As depicted in Table 1, small-sized and service firms dominated the firms under study at 58.7% and 56.9%, respectively. Firms studied had been in operation for at least five years, with the majority having 5–10 years’ experience, representing 38.2% of the firms.

**Table 1:** Firms characteristics.

Firms and respondents' background	Frequency	Percentages (%)
Industry	685	100.00
Manufacturing	295	43.1
Service	390	56.9
Size	685	100.00
6-29 employees	402	58.7
30-99 employees	283	41.3
Age of firm	685	100.0
5–10 years	262	38.2
11–15 years	208	30.4
15–20 years	118	17.2
Above 20 years	97	14.2

### 3.2 Measurements

The questionnaires were made up of items measuring management commitment (MC), customer integration (CIN), supplier integration (SI), internal integration (INT), government support (PGS), and innovation performance (IP). The various constructs’ reliability, validity, and internal consistency have been validated in studies such as [13,[26], [29],[46], [47],[75], [94]]. We examined all the study items on a 7-point Likert scale, comprising of 1 (strongly disagree), 2 (mostly disagree), 3 (slightly disagree), 4 (neutral), 5 (slightly agree), 6 (mostly agree), and 7 (strongly agree). We used the 7-point Likert scale because it offers respondents more choices than the 5-point Likert scale and is used mainly in supply chain management studies.

#### 3.2.1 Management commitment

Regarding this study, management commitment (MC) refers to top managers who establish and communicate the firm's vision and goals, involve and participate in the firm's management, allot resources and time, and motivate and empower employees to internalize firm values and monitor operations [13,14,37].

To measure the commitment level of SME top managers, five items were adapted from the study [13]. The scale assessed managers' commitment and support level, producing a Cronbach's alpha (α) value of 0.872. A sample item for this study included: “The positive attitude of management to solve problems concerned with employees.” Our MC scale in this study yielded an α = 0.873, signifying high internal consistency. All the items of the variables were assessed on a seven-point Likert scale of 1 (strongly disagree) to 7 (strongly agree).

#### 3.2.2 Supply chain integration

The supply chain integration scale contained CIN, SI, and INT dimensions. The CIN and SI were measured with 12 items each, and INT had nine items adapted from Refs. [24,[94], [95]]. In the study of [[94], [95]], CIN, SI, and INT yielded α = 0.8423, 0.8885, and 0.9363, respectively. Furthermore, the [24] scale brought forth α = 0.92, 0.94, and 0.92 concerning CIN, SI, and INT. The Scales of the two studies depicted good internal consistencies. Sample questions for each of the three dimensions are: “There is linkage with customers through information network” (CIN), “Information exchange with our major suppliers through information network” (SI), and “Data integration among internal functions” (INT). In this current study, CIN, SI, and INT gave an α = 0.954, 0.918, 0.852, respectively. These results illustrate that the three scales had high internal consistency, and the data was fit for further analysis. The various variables’ items were assessed on a seven-point Likert scale of 1 (strongly disagree) to 7 (strongly agree).

#### 3.2.3 Government support

[[26], [27]] developed the government support (PGS) scale. The scale showed high internal consistency and detailed an α = 0.71. A more recent study by Ref. [[28], [29]] adapted the same scale and adjusted the items to 5, yielding an α = 0.89. An example item is: “The government implemented policies and programs that have been beneficial to our firm's operations.” In our current study, researchers used the original four items scale of PGS [[26], [27]]. The 4 items scale produced α= (0.832) and a construct reliability value of (0.833), depicting an excellent internal consistency. All the items of this variable were assessed on a seven-point Likert scale of 1 (strongly disagree) to 7 (strongly agree).

#### 3.2.4 Innovation performance

Innovation performance (IP) is the dependent variable of the study. IP scale was measured with 4 items adopted from Ref. [[46], [47]]. In Ref. [[46], [47]] study, IP scale recorded α, AVE, and CR values of 0.824, 0.633, and 0.873, respectively. An example question is: “We are able to develop new products or services with speed.” In this current study, IP scale yielded α, AVE, and CR values of .830, 0.563, and 0.834, respectively, depicting a high level of internal consistency. The study concentrated on firm-level innovation, and all the variable items were assessed on a seven-point Likert scale of 1 (strongly disagree) to 7 (strongly agree).

#### 3.2.5 Control variables

In the analysis, variables such as the firm's (age, size, and industry) were employed as control variables. These control variables were chosen because they have been shown to have unique characteristics influencing SMEs' innovation performance [[46], [47]].

### 3.3 Ethical consideration

Before collating this study's data, we obtained ethical approval from Ghana Enterprise Agency (GEA). Besides the selected small and medium enterprises (SMEs) ethics, internal leaders also consented and supported the data collection. All the respondents voluntarily participated and were made aware of the study's aim. Additionally, they received guarantees of complete confidentiality and anonymity for their responses. The respondents were informed that their responses would only be used for academic purposes.

### 3.4 Data analysis

We utilized the statistical package for social science (SPSS) V. 26 and the analysis of moment structure (Amos) V. 26 statistical tools to evaluate the data. To ascertain that the data fit well, confirmatory factor analysis (CFA), was conducted. Again, AMOS was utilized to ensure that the data suffer not from negative indeterminacy. Hence, choosing AMOS over other SEM techniques increased our confidence in the data's accuracy and appropriateness. Our data did not exhibit any inadmissible solutions in general. Again, the validity and construct reliability of the variables were estimated using the AMOS plugin developed by Ref. [[95], [96]].

The study deleted four items measures (three items from the INT and one from SI) during the CFA analysis. This approach of deleting items improves the data's model fit, and other indicators like the composite reliability and average variance extracted and is consistent with [[96], [98]]. Hence, INT7, INT8, INT9 and SI4 were deleted (factor loadings< 0.50). Inter-factor correlation analysis was assessed using SPSS V. 23 to provide preliminary insights on the hypotheses.

## 4 Results

### 4.1 Common method variance (CMV)

As emphasized by Refs. [99,100], while conducting firm-level analysis, a single respondent is chosen to answer a questionnaire on behalf of the firm, which could lead to potential CMV. Hence, we applied several techniques described by Ref. [101] to control CMV. First, we randomized the research questionnaire's order. Again, we explained to the respondents that the study was exclusively conducted for academic purposes and that the responses would be held in strict confidence. Also, we urged the respondents to answer the questions honestly and that there were no wrong or correct answers. We performed Harman's single-factor test to further check CMV by loading the entire research items onto a single factor. Harman's single test results revealed one factor with an eigenvalue of 11.197, accounting for 26.038% of the total variance. The variance is less than the 50% threshold proposed in previous studies [[46], [47],^102^]. The result suggests that a single factor could not explain more than 50% of the variance in our data set, indicating that CMV was not an issue in this current study.

### 4.2 Reliability, validity, and unidimensionality of the variables

Table 2 illustrates the six investigated variables' reliability, validity, and unidimensionality. As suggested by Ref. [103], unidimensionality aids researchers in identifying the set of measurements that underpin a single construct. A comparative fit index (CFI) of 0.90 or above, according to Ref. [104], offers considerable evidence for unidimensionality. However [[95], [98]], advocated a combination of CFI = 0.95 or more and standardized root mean square residual (SRMR) = 0.06 or less to establish unidimensionality. Hence, this study's six tested variables produced SRMR values lower than 0.06 and CFI values higher than 0.95. Thus, the data of the survey achieved unidimensionality. Furthermore, in the CFA factor loading results, all standardized beta values were greater than 0.60, suggesting significance at a 95% confidence interval. The entire model fit for the measures also achieved CFA values of X^2^ = 1480.009, X^2^/df = 1.751, root means square error of approximation (RMSEA) = 0.033, Goodness of Fit Index (GFI) = 0.909, SRMR = 0.036, Tucker–Lewis Fit Index (TLI) = 0.960, CFI = 0.962, specifying that the study model fits the data satisfactorily.

**Table 2:** Factor loadings, reliability and validity analysis.

Construct	Indicators	β-values	t-values	CFI	SRMR	α	CR	AVE
management Commitment (MC)	MC5	.914	–	0.994	0.020	.873	0.875	0.586
	MC2	.768	25.174					
	MC1	.757	24.563					
	MC3	.701	21.754					
	MC4	.665	20.135					
Customer integration (CIN)	CIN6	.904	–	0.984	0.021	.954	0.954	0.636
	CIN10	.847	32.318					
	CIN11	.825	30.593					
	CIN1	.814	29.716					
	CIN3	.803	28.901					
	CIN9	.778	27.261					
	CIN7	.779	27.270					
	CIN2	.774	26.948					
	CIN12	.781	27.441					
	CIN4	.776	27.082					
	CIN5	.738	24.802					
	CIN8	.732	24.459					
Supplier integration (SI)	SI9	.909	–	0.990	0.024	.918	0.918	0.509
	SI3	.757	25.468					
	SI8	.737	24.318					
	SI6	.723	23.519					
	SI7	.700	22.345					
	SI10	.727	23.744					
	SI1	.694	22.025					
	SI11	.659	20.374					
	SI12	.669	20.842					
	SI2	.649	19.926					
	SI5	.574	16.750					
Internal integration (INT)	INT4	.773	–	0.994	0.023	.852	0.853	0.501
	INT2	.722	18.413					
	INT3	.697	17.729					
	INT6	.728	18.566					
	INT5	.661	16.751					
	INT1	.619	15.625					
Government support (PGS)	PGS4	.780	–	0.996	0.008	.832	0.833	0.555
	PGS2	.786	19.180					
	PGS1	.703	17.397					
	PGS3	.707	17.497					
Innovative performance (IP)	IP1	.942	–	0.995	0.021	.830	0.834	0.563
	IP3	.651	18.561					
	IP2	.660	18.881					
	IP4	.712	20.791					

Notes. a = Cronbach alpha; b = Standardized factor loadings Abbreviations: CFI, Comparative fit index; SRMR, Standardized root mean square residual; CR, Construct reliability; AVE, Average variance extracted.

Although establishing unidimensionality is significant, it does not entirely validate the usefulness of the measures [[98], [102],[104], [105]]. Therefore, it is appropriate to perform a reliability analysis to establish the construct reliability of the measures. Each measure's construct reliability coefficients varied from 0.830 to 0.954, suggesting high internal consistency. The test achieved high convergent validity with AVE values ranging from 0.501 to 0.636 for the various variables. The discriminant validity of the various constructs was evaluated by comparing the squared root of the AVEs with their inter-correlation values displayed along the diagonal line of the inter-factor correlation matrix. Table 3 results indicated that the square root of the AVEs was higher in all cases, confirming that discriminant validity exists among the various constructs and are unique to each other. To further verify our study's discriminant validity, we run a heterotrait-monotrait (HTMT) ratio of correlation analysis. As specified in Table 4, Our results presented HTMT values of less than 0.85, as indicated by Ref. [106]. This further proves that our measures were related but distinct from each other.

**Table 3:** Mean, standard deviation, correlations analysis, and discriminant validity.

	MEAN	SD	CIN	SI	MC	INT	PGS	IP									
Size	1.413	.493	–														
Age	2.073	1.057	–														
Industry	1.431	.496	–														
CIN	3.194	1.223	***0.797***	–													
SI	3.124	1.015	0.266***	***0.713***													
MC	3.142	1.065	0.316***	0.280***	***0.765***												
INT	3.557	1.087	0.272***	0.336***	0.279***	***0.707***											
PGS	2.982	1.268	0.202***	0.202***	0.201***	0.257***	***0.745***										
IP	3.306	1.214	0.319***	0.344***	0.333***	0.293***	0.276***	***0.750***									

Notes. The diagonal values (in bold) are the square root of the average variance extracted; ***p < .001; Discriminant validity values are reported in italics. Abbreviations: MC, Management commitment; CIN, Customer integration; SI, Supplier integration; INT, Internal integration; PGS, Government support; IP, Innovative performance.

**Table 4:** Heterotrait-monotrait ratio of correlation.

HTMT results for the discriminant validity					
	IP	MC	PGS	INT	SI
IP	–				
MC	0.338				
PGS	0.275	0.192			
INT	0.357	0.312	0.531		
SI	0.345	0.274	0.228	0.359	
CIN	0.32	0.317	0.359	0.325	0.266

Note. IP, Innovation performance; MC, Management commitment; PGS, Government support; INT, Internal integration; SI, Supplier integration; CIN, Customer integration.

### 4.3 Means, standard deviation, and correlation analysis

Table 3 shows the measurements’ mean, standard deviation, and inter-factor correlation analysis. The correlation analysis outcomes depict the MC (independent variable) significantly correlated with innovation performance (dependent variable) and SCI, that is, customer, internal, and supplier (mediator variables), thereby indicating preliminary support for H1 and H2a, H2b, H2c. Again, SCI positively correlates with innovation performance, suggesting some initial support for H3.

### 4.4 Hypotheses testing

#### 4.4.1 Main effect and mediating effects

We implored the hierarchical regression technique, run with SPSS version 26, to assess the various hypotheses advanced for the study's main effect, mediating and moderating effects, while controlling for the firm's size, age, and industry. The results are recorded in Table 5, Table 6, Table 7. First, the results in model 2 in Table 5, Table 6, Table 7 showed that MC positively influenced SMEs' innovation performance. Therefore, H1 is supported. Second, Model 3 in Table 5, Table 6, Table 7 also revealed that MC significantly influenced INT, CIN, and SI. Thus, H2a, H2b, and H2c were also supported. Third, SCI (INT, CIN, SI) was engaged as an independent variable in Table 5, Table 6, Table 7, as reported in Model 4 of each table. The results indicated that all dimensions of SCI positively and significantly predicted SMEs' innovation performance. Thus, H3a, H3b, and H3c were supported. Last, for evaluating hypotheses H4a, H4b, and H4c, which are about the mediation role of SCI dimensions (INT, CIN, SI) between MC and SMEs' innovation performance, we relied on a technique suggested by Ref. [107]. The technique emphasizes regression weights and correlation of the studied variables, and four criteria must be met for full or partial mediation support [107]. First, the independent variable (MC) should significantly affect the mediator variable, SCI (INT, CIN, SI). Again, MC should significantly affect the dependent variable (SMEs' innovation performance). Furthermore, the mediator should significantly influence the dependent variable. Finally, to achieve full mediation, the direct effect between the independent and dependent variables must be insignificant when the mediator is introduced to the relationship. In the case of partial mediation, the direct effect between the independent and dependent variables remains significant when the mediator variable is introduced in the regression equation. Hence, as shown in model 5 in Table 5, Table 6, Table 7, we regressed SMEs' innovation performance on MC and SCI (INT, CIN, SI). The outcome indicated that MC significantly influenced SMEs' innovation performance. Again, SCI (INT, CIN, SI) also significantly impacted SMEs' innovation performance. Hence, the relationship between MC and SMEs innovation performance was partially mediated by all three dimensions of SCI. Thus, H4a, H4b, H4c were supported.

**Table 5:** Mediating role of internal integration in the relationship between management commitment and innovation performance.

Variable	IP	IP	INT	IP	IP
	Model 1 *β*(p-value)	Model 2 *β*(p-value)	Model 3 *β*(p-value)	Model 4 *β*(p-value)	Mode 5 *β*(p-value)
Constant	3.699 (.000)	2.605 (.000)	2.567 (.000)	2.721 (.000)	2.051 (.000)
Size	−.066 (.084)	−.029 (.431)	.021 (.575)	−.062 (.091)	−.033 (.361)
Age	.046 (.224)	.042 (.251)	−.002 (.958)	.046 (.215)	.043 (.239)
Industry	−.078 (.041)	−.075 (.041)	.030 (.413)	−.085 (.022)	−.081 (.025)
MC		.270 (.000)	.260 (.000)		.219 (.000)
INT				.249 (.000)	.193 (.000)
R^2^	.013	.084	.067	.075	.119
ΔR^2^	.013	.071	.066	.062	.106
F	2.931 (0.33^b^)	15.611 (.000^c^)	12.280 (.000^c^)	13.735 (.000^c^)	18.320 (.000^c^)

Note. β = Standardized beta coefficient, Values in parentheses represent p-values, *p < .05. **p < .01. ***p < .001, MC, Management commitment; INT, Internal integration.

**Table 6:** Mediating role of customer integration in the relationship between management commitment and innovation performance.

Variable	IP	IP	CIN	IP	IP
	Model 1 *β*(p-value)	Model 2 *β*(p-value)	Model 3 *β*(p-value)	Model 4 *β*(p-value)	Mode 5 *β*(p-value)
Constant	3.699 (.000)	2.605 (.000)	2.299 (.000)	2.776 (.000)	2.135 (.000)
Size	−.066 (.084)	−.029 (.431)	−.039 (.295)	−.045 (.221)	−.021 (.558)
Age	.046 (.224)	.042 (.251)	.066 (.072)	.028 (.454)	.029 (.428)
Industry	−.078 (.041)	−.075 (.041)	−.050 (.167)	−.064 (.083)	−.065 (.073)
MC		.270 (.000)	.291 (.000)		.210 (.000)
CIN				.267 (.000)	.206 (.000)
R^2^	.013	.084	.097	.083	.122
ΔR^2^	.013	.071	.083	.070	.109
F	2.931 (.033^b^)	15.611 (.000^c^)	18.330 (.000^c^)	15.353 (.000^c^)	18.932 (.000^c^)

Note. β = Standardized beta coefficient, Values in parentheses represent p-values, *p < .05. ***p < .001. MC, Management commitment; CIN, Customer integration.

**Table 7:** Mediating role of supplier integration in the relationship between management commitment and innovation performance.

Variable	IP	IP	SI	IP	IP
	Model 1 *β*(p-value)	Model 2 *β*(p-value)	Model 3 *β*(p-value)	Model 4 *β*(p-value)	Mode 5 *β*(p-value)
Constant	3.699 (.000)	2.605 (.000)	2.694 (.000)	2.460 (.000)	1.825 (.000)
Size	−.066 (.084)	−.029 (.431)	−.048 (.200)	−.042 (.251)	−.018 (.625)
Age	.046 (.224)	.042 (.251)	−.035 (.347)	.055 (.129)	.051 (.157)
Industry	−.078 (.041)	−.075 (.041)	−.035 (.350)	−.067 (.066)	−.067 (.062)
MC		.270 (.000)	.247 (.000)		.210 (.000)
SI				293 (.000)	.242 (.000)
R^2^	.013	.084	.069	.098	.139
ΔR^2^	.013	.071	.060	.085	.126
F	2.931 (.33^b^)	15.611 (000^c^)	12.606 (.000^c^)	18.471 (.000^c^)	21.848 (.000^c^)

Note. β = Standardized beta coefficient, Values in parentheses represent p-values, *p < .05. 01. ***p < .001. MC, Management commitment; SI, Supplier integration.

#### 4.4.2 Moderation effects of government support

**Fig. 2:**
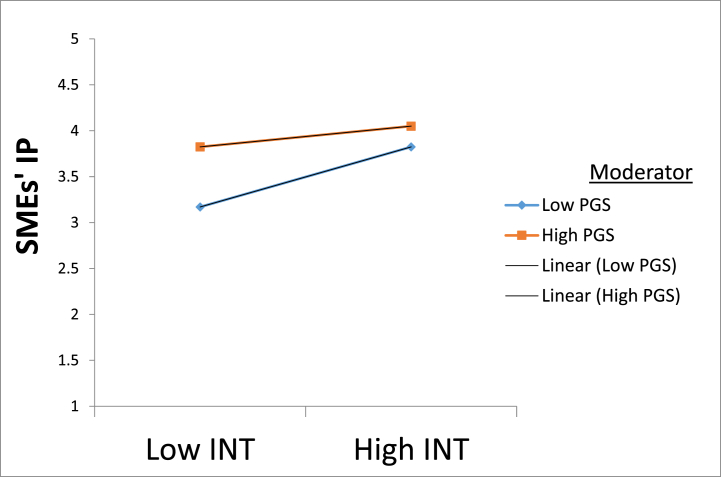
A graph of the moderating impact of government support (PGS) in the relationship between internal integration (INT) and innovative performance (IP). Note. INT, Internal integration; SMEs' IP, Small and medium enterprises innovation performance.

**Fig. 3:**
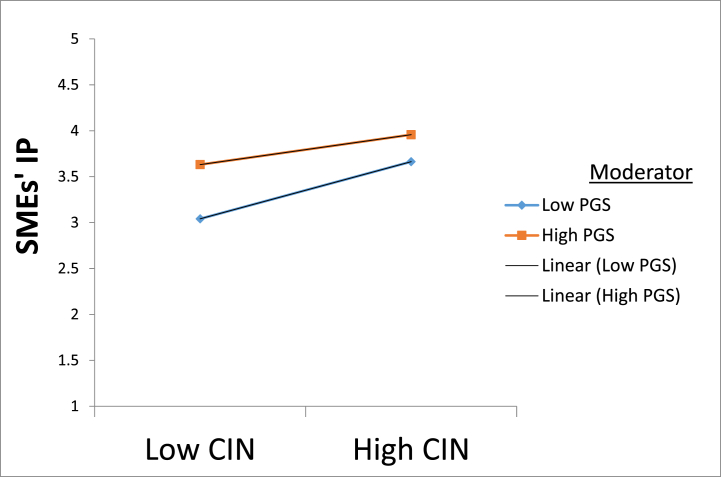
A graph of the moderating influence of government support (PGS) in the relationship between customer integration (CIN) and innovative performance (IP). Note. CIN, Customer integration; SMEs' IP, Small and medium enterprises innovation performance.

**Fig. 4:**
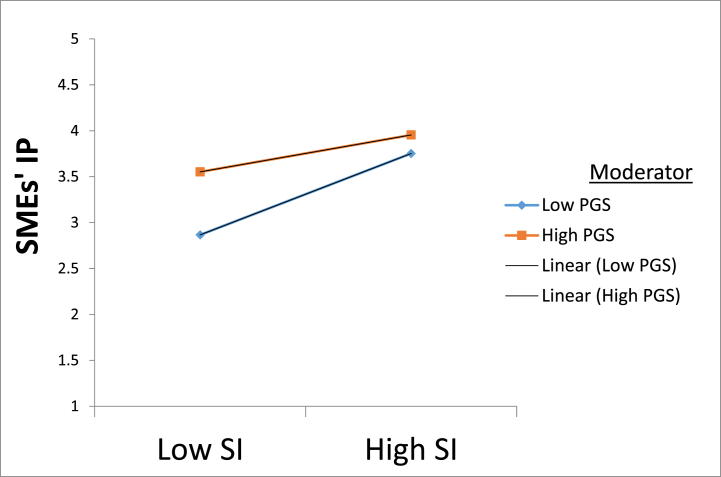
A graph of the moderating impact of government support (PGS) in the relationship between supplier integration (INT) and innovative performance (IP). Note. SI, supplier integration; SMEs' IP, Small and medium enterprises innovation performance.

Researchers utilized hierarchical regression to assess the moderating effects of government support (PGS) in the predictive effects of supply chain integration (SCI) on SMEs' innovation performance. We evaluated the three dimensions of SCI variables: internal, customer, and supplier integration as independent variables and PGS as a moderating variable. Before examining the moderating analysis, PGS, internal, customer, and supplier integration were mean-centered to reduce multi-collinearity. In Table 8, the outcome estimated in models 2, 4, and 6 showed that the three dimensions of SCI still had a significant positive impact on SMEs' innovation performance after being centralized. This further verified H3. Models 3, 5, and 7 in Table 8 depict that PGS significantly and negatively moderated the relationship between SCI (internal, customer, supplier) and SMEs' innovation performance. Though the interactions of PGS and SCI components on innovation performance were significant, their negative impacts contradict the study's hypotheses for the moderation effects. Therefore, H5a, H5b, and H5c were not supported. A graphical representation of the results can be found in Fig. 2, Fig. 3, Fig. 4.

**Table 8:** Moderating effects of government support in the relationship between Supply chain integration (internal, customer, supplier) and innovation performance.

Variables	SMEs Innovation performance						
	Model 1 *β(*p-value)	Model 2 *β(p-value)*	Model 3 *β(p-value)*	Model 4 *β(p-value)*	Model 5 *β(p-value)*	Model 6 *β(p-value)*	Model 7 *β(p-value)*
Constant	3.699 (.000)	3.693 (.000)	3.717 (.000)	3.615 (.000)	3.573 (.000)	3.556 (.000)	3.531 (.000)
size	−.162 (.084)	−.138 (.122)	−.123 (.165)	−.101 (.256)	−.083 (.349)	−.093 (.290)	−.063 (.471)
Age	.053 (.224)	.042 (.314)	.036 (.382)	.024 (.568)	.025 (.538)	.051 (.210)	.053 (.187)
industry	−.191 (.041)	−.195 (.028)	−.196 (.026)	−.151 (.088)	−.127 (.149)	−.157 (.072)	−.154 (.074)
INT		.228 (.000)	.220 (.000)				
CIN				.227 (.000)	.237 (.000)		
SI						.309 (.000)	.322 (.000)
PGS		.195 (.000)	.220 (.000)	.199 (.000)	.221 (.000)	.197 (.000)	.222 (.000)
INT*PGS			−.107 (.001)				
CIN*PGS					−.074 (.003)		
SI*PGS							−.121 (.000)
R^2^	.013	.114	.129	.125	.136	.139	.161
ΔR^2^	.013	.102	.116	.112	.123	.126	.148
F	2.931 (.033^b^)	17.528 (.000^c^)	16.714 (.000^c^)	19.340 (.000^C^)	17.788 (.000^C^)	21.890 (.000^C^)	21.626 (.000^C^)

Notes. β = Unstandardized beta coefficient, Values in parentheses represent p-values, ***p < .001, **p < .01, *p < .05. INT, Internal integration; CIN, Customer integration; SI, Supplier integration; PGS, Government support; INT*GS, Internal integration x Government support; CIN*GS, Customer integration x Government support; SI*PGS, Supplier integration x Government support.

## 5 Discussion

This study relied on the dynamic capability view (DCV) to explore the direct and indirect effects of management commitment (MC) on SMEs' innovation performance through supply chain integration (SCI), (internal, customer, supplier) as a mediator. Again, the study employed government support (PGS) to moderate the predictive effects of SCI on SMEs’ innovation performance. The outcome from the hierarchical regression analysis presented a strong foundation for the hypothesized relationships.

First, we hypothesized that MC significantly affects SMEs' innovation performance (H1), endorsed by the analysis, thereby confirming earlier similar findings [13,14,[81], [82]]. The result implies that committed managers are vital in enhancing SMEs' innovation performance. The study demonstrates that dynamic capabilities are partly vested in managers or management teams [[59], [60]]. These developed capabilities aid managers initiate innovative ideas to detect and explore opportunities that tend to yield higher innovation performance. Thus, committed managers with solid dynamic capabilities will support the firm's vision and goals to allocate resources strategically, apply established policies, and empower the employees to attain competitive advantage [49,108].

Second, our findings revealed that MC had a significant positive influence on the dimensions of SCI (internal, customer, supplier) (H2a, H2b, and H2c), confirming findings in similar studies such as [25,37]. The results establish the importance of managers' attitudes, support, and motivation toward achieving effective SCI. Also, the study of [17] emphasized that employees' commitment benefits SCI activities. Last [25], endorsed these prepositions by suggesting that SCI depends on people's motivations and attitudes. Hence, from the standpoint of DCV, committed managers will desire to implement established policies, allot resources, and empower employees to integrate the firm's internal systems to ascertain customer and supplier integration via information sharing and collaborate in strategic decision-making.

Third, SCI (internal, customer, supplier) significantly influenced SMEs' innovation performance (H3a, H3b, and H3c). These findings are supported by studies such as [[20], [21], [22]]. The result emphasizes that effective SCI produces higher performance. Internal integration (INT), identified as a firm's internal capability, is a precondition for external integration [56]. Therefore, SMEs' with well-integrated and synchronized structures improve innovation performance.

Again, external integration (customer, supplier) denotes a higher level of SCI [24]. More so, the results of (H3b and H3c) endorse the proposition that external integration (customer, supplier) is a critical component that fosters a firm's commitment to its suppliers and customers [21] which helps in improving firm performance. Thus, this demands that the focal firm form an alliance and maintain regular contact with external partners such as retailers, wholesalers, and manufacturers, which could reduce transactional costs [57].

Fourth, our study's findings further indicated that all three dimensions of SCI partially mediate the MC effect on SMEs' innovation performance, endorsing H4a, H4b, and H4c. The results suggest that MC plays a vital role in improving SMEs' innovation performance by establishing a robust SCI. The mediation role of SCI in the relationship between MC and innovation performance endorses the results of [[16], [17],[43], [44]]. More so, the result is consistent with previous studies that suggest that MC is critical for the success of SCI [[24], [25],[69], [70]]. Therefore, it is evident that MC becomes the basis of competitive advantage for the firm and SCI. This study endorses these results by establishing MC as an intangible resource [41] benefits SCI and, through SCI, improves SMEs' innovation performance. Last, from the perspective of DCV, the result indicates that committed managers sense, seize and reconfigure strategic resources to implement robust SCI [36], further improving SMEs' innovation performance.

Last, surprisingly, the outcome of the moderation results failed to meet our expectations. Our expectations were unmet because PGS significantly and negatively moderated SCI (internal, customer, supplier) and innovation performance relationships. Though the negative interaction term did not meet our expectations, the results are worthy of concern. The results illustrate that due to the negative effect of PGS, at high levels of the PGS, the effects of SCI (internal, customer, supplier) on SMEs' innovation performance is weaker, while at lower levels of PGS, the effect of SCI (internal, customer, supplier) on SMEs' innovation performance is stronger. Our moderation results contradict the study findings of [[28], [29]] in which PGS assumed a positive moderation effect, further strengthening the innovative climate's positive impact on department innovations. The possible reason that could account for our study finding is an inadequate collaboration among policy implementors and other stakeholders, such as beneficiaries of the PGS (SMEs') and local service agencies who are to ensure the effectiveness of the policy implementation [109]. This could lead to gaps in the accessibility of the PGS policy, such as technical training, credit, loans, services, tax waivers, network building, research, and development, leading to the ineffectiveness of the policy. Generally, the study was carried out during and after the COVID-19 era, which disrupted most economies, leaving the supply chain incapacitated and resulting in a severe financial burden on firms [[91], [92]], especially SMEs'. The financial burden assumed by the SMEs' due to low productivity could encourage firms to re-channel acquired PGS in the form of finance to offset their operational, creditors, and wage bills.

## 6 Theoretical contribution

The study makes numerous theoretical contributions. First, thus, by assessing the effects of management commitment (MC) on small and medium enterprises (SMEs’) innovation performance, the current study reveals the vital role of intangible resources such as commitment play in aiding firms to achieve higher innovation performance.

Second, by espousing MC influence on the individual dimensions of supply chain integration (SCI), (internal, customer, supplier), the study's results enrich the dynamic capability view (DCV). The result signifies the significant role committed managers have to play in achieving effective SCI.

Third, researchers presented the dimensional mediation effects of SCI (internal, customer, supplier) in the relationship between MC and SMEs' innovation performance. Studies such as [16,[69], [70]] have attempted to clarify the mediation role of SCI in the relationship between employee commitment and firm performance. Thus, this current study extends the relationship by assessing the mediation role of SCI in the relationship between management commitment and innovation performance from the perspective of SMEs'. Hence, from the perspective of DCV, we enrich the literature on SCI by assessing the antecedents of SCI from the perspective of intangible resources (management commitment) and endorsing SCI signal effect on SMEs’ innovation performance.

Fourth, researchers such as [[21], [22],[66], [67]] have categorized SCI dimensions as (internal, customer, and supplier) and assessed their impact on firm performance. Therefore, from the perspective of DCV, our results reaffirm the significance of SCI in reducing production cost, improving product quality, shortening cycle time, increasing response rates, and improving customer satisfaction [24].

Fifth, the study extends the relationship between SCI (internal, customer, supplier) and SMEs' innovation performance with a moderating variable, government support (PGS). Even though PGS has been tipped to upsurge a sustainable competitive performance in emerging economies [110], in the present study PGS failed to significantly moderate the relationship between SCI (internal, customer, supplier) and SMEs' innovation performance. This study's outcome suggests that PGS significantly enhances SMEs' innovation performance but PGS's interaction with SCI ((internal, customer, supplier) failed to positively and significantly influence SMEs' innovation performance.

Hence, our study contributes to the literature on SMEs’ innovation performance, SCI, and PGS.

### 6.1 Managerial implications

The managerial implications of this study are extremely valuable and crucial for businesses. Our study's managerial implications include: first, small and medium enterprises (SMEs) must prioritize developing commitment by implementing high work practices such as job security, job-based skill training, upgrading system, formal performance management, and work teams. These practices help upsurge skills and motivate and empower, which is pivotal in building management commitment [25].

Second, firms need to prioritize management commitment (MC) and promote supply chain integration (SCI), (internal, customer, supplier) because it is envisaged that a committed workforce will be more supportive of promoting strategic integration with supply chain partners. Firms can surge management commitment by building congruency, exciting work, clarity of purpose, feedback, fairness, empowerment, and autonomy.

Third, managers need to continuously integrate the firm's internal structures and link up with external partners to ascertain all benefits of SCI. For example, SCI can reduce production costs, shorten cycle time, enhance product quality, increase the response rate, and improve customer satisfaction [24].

Fourth, firms must pay attention to the human capital element of their organization to upsurge their managers’ commitment level and build effective SCI to achieve higher performance. Firms could ascertain higher innovation performance by improving their MC and SCI.

Fifth, managers must acknowledge that government support (PGS), a strategic external resource, could yield negative results if poorly implemented. Hence, sustainability must be built from the firm's internal structures through knowledge creation, uncertainty and knowledge management, organizational intelligence, efficient supply chain integration, and robust financial framework [111].

Last, to ensure the robustness and effectiveness of a designed policy such as PGS, policy implementers must track the delivery process, identify emerging obstacles, and build up additional resources where necessary to tackle specific problems [109]. Again, it is acknowledged that tracking performance delivery alone is not sufficient to achieve effective implementation, particularly where the policy is complex and in an uncertain environment. Therefore, [112] argued that frontline policy implementers who are more equipped with the challenges of the delivery process must be engaged to tap into their perceptions and experiences to shape the implementation process continuously.

## 7 Limitations and suggestions for further research

This study has some drawbacks. First, the study focused on manufacturing and service SMEs only, of which there are several sectors in Ghana enterprise agencies. Hence, future studies must capture other sectors for easy generalization.

Second, the study relied on the perception of general managers. We did not assess the perception of lower employees. Hence, a likely area for future research is investigating employees' perceptions of how employees' commitment impact SMEs’ innovation performance within different firm settings such as large firms.

Third, the current study assessed the model in the developing economy of Ghana, which may not be considered a good representation worldwide. Thus, additional evidence could be gathered from other emerging economies and developed markets to ascertain more valuable insights.

Fourth, the study did not consider gender, age, and location spread as controls. Future studies should consider these controls so as establish the entrepreneurs' personalities which are generally affected by SMEs’ for easy generalization.

Last, we gathered the research data with a cross-sectional design. Future studies could consider a longitudinal design that might aid in drawing a firmer conclusion on the relationship between MC and SMEs’ innovation performance, the mediation effects of SCI, and the moderating role of PGS at the firm level.

## Author contribution statement

Hao Chen: Timothy Amoako, Collins Ewudzie Quansah, Stephen Abiam Danso, Dawud Jidda Jidda: Conceived and designed the experiments; Performed the experiments; Analyzed and interpreted the data; Contributed reagents, materials, analysis tools or data; Wrote the paper.

## Data availability statement

Data will be made available on request.

## Additional information

Supplementary content related to this article has been published online at [URL].

## Declaration of competing interest

The authors declare no conflict of interest.
