# Supplementary material for: Assessment of the impact of management commitment and supply chain integration on SMEs’ innovation performance: Moderation role of government support
Source: Heliyon. 2023 Apr 29;9(5):e15914. doi: 10.1016/j.heliyon.2023.e15914 (PMC10195914; doi:10.1016/j.heliyon.2023.e15914)
Supplement: Multimedia component 1 [file mmc1.docx]

**APPENDIX**

Measurement of the research constructs

**INDEPENDENT VARIABLE**

**Perceived management commitment**

A: Show the extent to which management commit themselves to the firm. Please tick appropriately and note that: 1= (strongly disagree), 2= (mostly disagree), 3= (slightly disagree), 4= (neutral), 5= (slightly agree), 6= (mostly agree) and 7= (strongly agree)

1 The positive attitude of management to solve problems concerned with employees.

2 Management support for implementing quality-related programs.

3 Management support middle management for quality performances.

4 Tower level managers conduct the daily meeting before starting the work.

5 Managers attend quality-related seminars and workshops

**MEDIATING VARIABLE**

Statement for Supply Chain Integration

The questions in this scale ask about your response to Supply Chain Integration of your Small and Medium Enterprise (SME). Please pay attention to your SME’s Supply Chain Integration that is Internal, Customer, and Supplier. Please choose your response to each statement by ticking most appropriate option.

Please note that:

1= (strongly disagree), 2= (mostly disagree), 3= (slightly disagree), 4= (neutral), 5= (slightly agree), 6= (mostly agree) and 7= (strongly agree)

B1: Please indicate the extent of integration or information sharing between your firm and major customers.

|  | STATEMENT |  | |  |  |  |  |
| --- | --- | --- | --- | --- | --- | --- | --- |
| 1 | There is linkage with our customers through information network. |  | |  |  |  |  |
| 2 | Computerization for our major customers ordering. |  | |  |  |  |  |
| 3 | Sharing of market information from our major customers. |  | |  |  |  |  |
| 4 | Communication with our major customers. |  | |  |  |  |  |
| 5 | The establishment of quick ordering systems with our major customers. |  | |  |  |  |  |
| 6 | Follow-up with our major customers for feedback. |  | |  |  |  |  |
| 7 | The frequency of period contacts with our major customers |  | |  |  |  |  |
| 8 | Our major customers share point of sales (POS) information with us |  | |  |  |  |  |
| 9 | Our major customers’ shares demand forecast with us |  | |  |  |  |  |
| 10 | We share our available inventory with our major customers |  | |  |  |  |  |
| 11 | We share our consignment plan with our major customers |  |  | |  |  |  |
| 12 | Our customers are actively involved in our product design process | | | | | | |

B2: Please indicate the extent of integration or information sharing between your firm and your major Supplier.

|  | STATEMENT |  |  |  |  |  |
| --- | --- | --- | --- | --- | --- | --- |
| 1 | Information exchange with our major suppliers through information networks. |  |  |  |  |  |
| 2 | The establishment of quick ordering systems with our major suppliers. |  |  |  |  |  |
| 3 | Strategic partnership with our major suppliers. |  |  |  |  |  |
| 4 | Stable procurement through network with our major suppliers. |  |  |  |  |  |
| 5 | The participation level of our major supplier in the process of procurement |  |  |  |  |  |
| 6 | The participation level of our major suppliers in the design stage |  |  |  |  |  |
| 7 | Our major suppliers share their production schedule with us |  |  |  |  |  |
| 8 | Our major suppliers share their production capacity with us |  |  |  |  |  |
| 9 | Our major suppliers share available inventory with us |  |  |  |  |  |
| 10 | We share our demand forecast with our major suppliers |  |  |  |  |  |
| 11 | We share our inventory levels with our major suppliers |  |  |  |  |  |
| 12 | We help our major suppliers to improve its process to better meet our needs |  |  |  |  |  |

B3: Please indicate the level of integration within your firm.

|  | STATEMENT |  |  |  |  |  |
| --- | --- | --- | --- | --- | --- | --- |
| 1 | Data integration among internal functions. |  |  |  |  |  |
| 2 | Enterprise application integration among internal functions. |  |  |  |  |  |
| 3 | Integrative inventory management. |  |  |  |  |  |
| 4 | Real-time searching of the level of inventory. |  |  |  |  |  |
| 5 | Real-time searching of logistics-related operating data. |  |  |  |  |  |
| 6 | The utilization of periodic interdepartmental meetings among internal functions. |  |  |  |  |  |
| 7 | The use of cross-functional teams in process development. |  |  |  |  |  |
| 8 | The use of cross-functional teams in new product development |  |  |  |  |  |
| 9 | Real-time integration and connection among all internal functions through sales and delivery |  |  |  |  |  |

**MODERATING VARIABLE**

**Government support**

C: To what extent do you agree with the following statements in relation to government support in your firm? Please tick appropriately and note that: 1= (strongly disagree), 2= (mostly disagree), 3= (slightly disagree), 4= (neutral), 5= (slightly agree), 6= (mostly agree) and 7= (strongly agree)

1 The government implemented policies and programs that have been beneficial to our firm’s operations.

2 Our firm enjoyed support, (that is financial, example, tax deductions and financial subsidies, skill training etc) from the government.

3 The government provided the needed technological information and technical support to our firm.

4 The government helped our firm to obtain licenses for imports of technology, manufacturing and other equipment.

**DEPENDENT VARIABLE**

**Innovation performance (IP):**

D: To what extent do you agree with the following statements related to the performance of the firm? Please tick appropriately and note that: 1= (strongly disagree), 2= (mostly disagree), 3= (slightly disagree), 4= (neutral), 5= (slightly agree), 6= (mostly agree) and 7= (strongly agree)

1 We are able to develop new products/services with speed

2 We are able to launch new products/services on time

3 Introduced a number of changes in our business processes in past one year

4 Quick response to the new processes introduced by competitors within our industry
